# All‐In‐One Thermal Protection: Multifunctional Synergy in Hierarchically Structured Dual‐Oxide Nanofiber Aerogel

**DOI:** 10.1002/advs.202516126

**Published:** 2025-11-21

**Authors:** Zijian Zhao, Shujing Li, Han Ma, Zhou Zhou, Wentao Zhao, Zichen Wei, Yanbin Li, Qingguo Fei, Yueming Sun, Yunqian Dai

**Affiliations:** ^1^ School of Chemistry and Chemical Engineering Southeast University Nanjing 211189 China; ^2^ School of Mechanical Engineering, Southeast University Southeast University Nanjing 211189 China; ^3^ Ministry of Education Key Laboratory of Structure and Thermal Protection for High‐Speed Aircraft Nanjing 211189 China

**Keywords:** aerogel, Al_2_O_3_, heat obstruction, nanofiber, SiO_2_, thermal insulation

## Abstract

Ceramic nanofiber aerogels are emerging as promising thermal protection materials, yet their brittleness remains a critical challenge. This work designs a hierarchical fibrous aerogel consisting of streamlined dual‐oxide nanofibers featuring a dense‐sheath/porous‐core architecture, simultaneously overcoming the intrinsic fragility while enabling thermal‐protection. The obtained aerogel demonstrates ultra‐low thermal conductivity and exceptional thermomechanical stability a high working temperatures. The aerogel structure, constructed from arbitrarily bendable nanofibers, reduces the overall density and effectively lowers thermal conductivity. The minimum density can reach as low as 8 mg·cm^−3^. This unique structure enhances the mechanical stability of the entire aerogel in a wide working temperature (−196 to 1300 °C) upon 80% compression. The thermal conductivity achieves an extremely low value of 7 mW·m^−1^·K^−1^. These attributes originate from the following mechanisms: depressing heat transfer by prolonging heat conduction pathways and offering interfacial insulation, while impeding phonon transport within nanopores. Upon simulated space‐ground integrated experiments at ≈1200 °C, the ceramic nanofiber aerogel exhibits remarkable resistance to thermal vibration. The nanofiber aerogel can serve as an all‐in‐one platform to synergistically integrate noise absorption and catalytic conversion, thereby paving the way for future practical applications.

## Graphical abstract

Through a cross‐scale structural design, a lightweight, heat‐resistant, and thermal‐insulating streamlined dual‐oxide nanofiber aerogel is prepared to achieve exceptional thermomechanical stability. Structural design of ceramic fibrous aerogel toward thermal protection, illustrating thermal insulation and stress dissipation. The streamlined dual‐oxide fibrous aerogel shows promising potential for a wide range of applications, from air to ground.

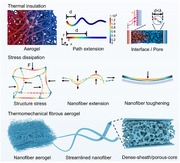

## 1 Introduction

Thermal‐protection systems of spacecraft safeguard delicate components from extreme temperatures during atmospheric or deep‐space missions,^[^ ^1^ ^]^ imposing comprehensive requirements on material performance and structure.^[^ ^2^, ^3^ ^]^ Nanofiber aerogels, leveraging their porosity and low density, have found extensive applications in aerospace, environmental filtration,^[^ ^4^, ^5^ ^]^ energy storage,^[^ ^6^ ^]^ tissue engineering,^[^ ^7^ ^]^ thermal management, and smart textiles,^[^ ^8^ ^]^ due to their porosity and low density. Traditional ceramic aerogels, however, suffer from harsh preparation conditions and high brittleness, making them prone to structural or functional failure under mechanical stresses or thermal shocks. In contrast, nanofibers offer significant advantages such as large surface area, interconnected pore structures, and lightweight properties.^[^ ^9^ ^]^ Thus, they are chosen as the fundamental building blocks for constructing high‐performance nanofiber aerogels. These aerogels overcome the limitations of traditional materials while retaining their inherent heat resistance.^[^ ^10^ ^]^ They have the potential to enhance thermal‐insulation efficiency in high‐temperature industrial processes, reduce energy consumption, and extend equipment lifespan, thereby positively impacting carbon emissions reduction.^[^ ^11^ ^]^ However, some existing nanofiber aerogels contain organic components or excessive elements, which may cause unexpected accidents under high temperatures or complex environments. Therefore, developing simple and effective ceramic nanofiber materials and structures tailored for various functional applications is of great significance for thermal protection. Such materials can address the issue of insufficient oxidation resistance and enable the full exploitation of thermal insulation advantages across multiple scales.^[^ ^12^, ^13^ ^]^

The flexibility and continuity of 3D nanofiber aerogels enhance material mechanical properties and thermal stability.^[^ ^14^ ^]^ These structures create robust, interconnected networks with complex paths. Traditional spinning technologies, however, suffer from low nanofiber production efficiency.^[^ ^15^ ^]^ They primarily produce 2D fiber membranes, which are further processed into 3D aerogels through cumbersome and energy‐consuming methods like freeze‐drying or 3D printing, with only linear thermal insulation performance improvement. As an alternative, high‐speed airflow drives micro/nanofiber spinning from solutions, effectively evaporating solvents to yield high‐quality dry fibers.^[^ ^16^ ^]^ The complex turbulent flow field facilitates nanofiber entanglement along intricate trajectories, forming 3D nanofiber aerogels.^[^ ^17^ ^]^ The emerging blow‐spinning technique enhances deposition rates and addresses efficiency issues. It allows one‐piece preparation of nanofiber aerogels, amplifying thermal insulation advantages. Combined with calcination and folding processes, it enables the preparation of nanofiber aerogels in various forms. Blow‐spinning's high efficiency, safety, and convenience support large‐scale production, meeting existing application demands and paving the way for new application areas.

In this work, we designed a streamlined dual‐oxide nanofiber featuring a dense‐sheath/porous‐core architecture to generate a lightweight nanofiber aerogel. The cross‐scale structure ensures structural and functional integrity under mechanical and thermal loads. This synergistic design improves the insulation and toughness of the entire fibrous aerogel. Assembled into a 3D network, they inherit and amplify fiber thermal insulation properties. The dual‐oxide aerogel achieves thermal conductivity as low as 7 mW·m^−1^·K^−1^. It performs exceptionally under high temperatures and maintains structural integrity across a wide working temperature (−196 to 1300 °C). This remarkable thermal‐stability makes the nanofiber aerogel suitable for extreme‐environment applications, as well as an all‐in‐one platform to synergistically integrate noise absorption and catalytic conversion.

## 2 Results and Discussion

### 2.1 Design of Streamlined Dual‐Oxide Fibrous Aerogel

The 3D network structure of the nanofiber aerogel, characterized by interconnected fibers and numerous pores, underpins its remarkable properties.^[^ ^18^ ^]^ During manufacturing, high porosity creates abundant space for air entrapment. As air is a poor heat conductor, this effectively impedes heat transfer—a fundamental principle of aerogels that contributes to exceptional thermal insulation.^[^ ^19^ ^]^ The low density arising from this porosity reduces weight, and lower mass generally equates to reduced heat‐carrying capacity. The complex network configuration also diminishes thermal convection. Additionally, the gas‐solid interface significantly complicates heat conduction in both solid and gas phases, resulting in remarkably low thermal conductivity. The interconnected network enhances stress distribution, and the sliding frame structure allows for mutual support and stress release under external forces, thereby improving mechanical strength.^[^ ^20^ ^]^ Consequently, this study focuses on designing and fabricating ultra‐lightweight oxide nanofiber aerogels for thermal insulation purposes.

Meanwhile, the superior properties of nanofiber aerogel are attributed to the nature of the nanofibers themselves and the porous architecture. Under the condition of equal displacement, the curved streamlined nanofibers are capable of furnishing longer propagation pathways for solid heat transfer.^[^ ^21^ ^]^ This unique geometric configuration of the nanofibers by blow spinning effectively increases the tortuosity of the heat transfer routes. Then, the composite structure with anisotropic bending influences the distribution characteristics within the material system.^[^ ^22^ ^]^ Both thermal transport and stress distribution benefit from this.^[^ ^23^ ^]^

The fabrication of streamlined nanofibers is based on skeletons featuring varying porosities, which present internal and external structural differences.^[^ ^24^ ^]^ Inspired by the hair structure of various animals in nature,^[^ ^25^ ^]^ including white tigers, polar bears,^[^ ^26^, ^27^ ^]^ camels,^[^ ^28^ ^]^ foxes, and so on. Inside, there is a delicate and intricate layered architecture. The outermost layer is covered by a tough and protective cuticle, while the inner layer consists of a soft, insulating, and resilient cortex that offers internal support. Mimicking the inner structure of the hair's composition, nanofibers are compact and air‐rich within their porous interior. The outer layer is made of high‐strength and abrasion‐resistant nanomaterials to cope with complex and variable external environments. The outer layer is encapsulated by a denser form of Al_2_O_3_, while the inner layer of SiO_2_ is loose and porous. This difference between the two layers endows the material with unique physical and chemical properties.^[^ ^29^ ^]^ One aspect is that, upon the heat flow traversing the two‐phase interface, heat contraction will lead to the generation of contact thermal resistance.^[^ ^30^ ^]^ The interfacial effect further impedes the transfer of heat.^[^ ^31^ ^]^ On the other hand, the thermal conductivity of Al_2_O_3_ is higher than that of SiO_2_, thereby creating a potential energy difference. It enables the heat accumulated on the nanofiber surface to quickly transfer out, in a manner analogous to water flowing downward. The synergistic effect enables superior heat dissipation and passive cooling.

Air molecules in the pores of the aerogel transfer heat by colliding with each other and with the walls. It is also known as the mobile conduction of phonons. Knudsen Effect's phenomenon illustrates the relationship between pores and gas thermal conductivity. Therefore, when the pore diameter is controlled to be smaller than the average free range of air molecules (i.e., 70 nm), the air thermal transfer can be effectively reduced.^[^ ^32^ ^]^ The design of reducing the size of the pores effectively inhibits the transmission of phonons between layers and scatters them on the surface. This significantly increases the thermal resistance and achieves a reduction in thermal conductivity and a favorable thermal insulation.

**Scheme 1:**
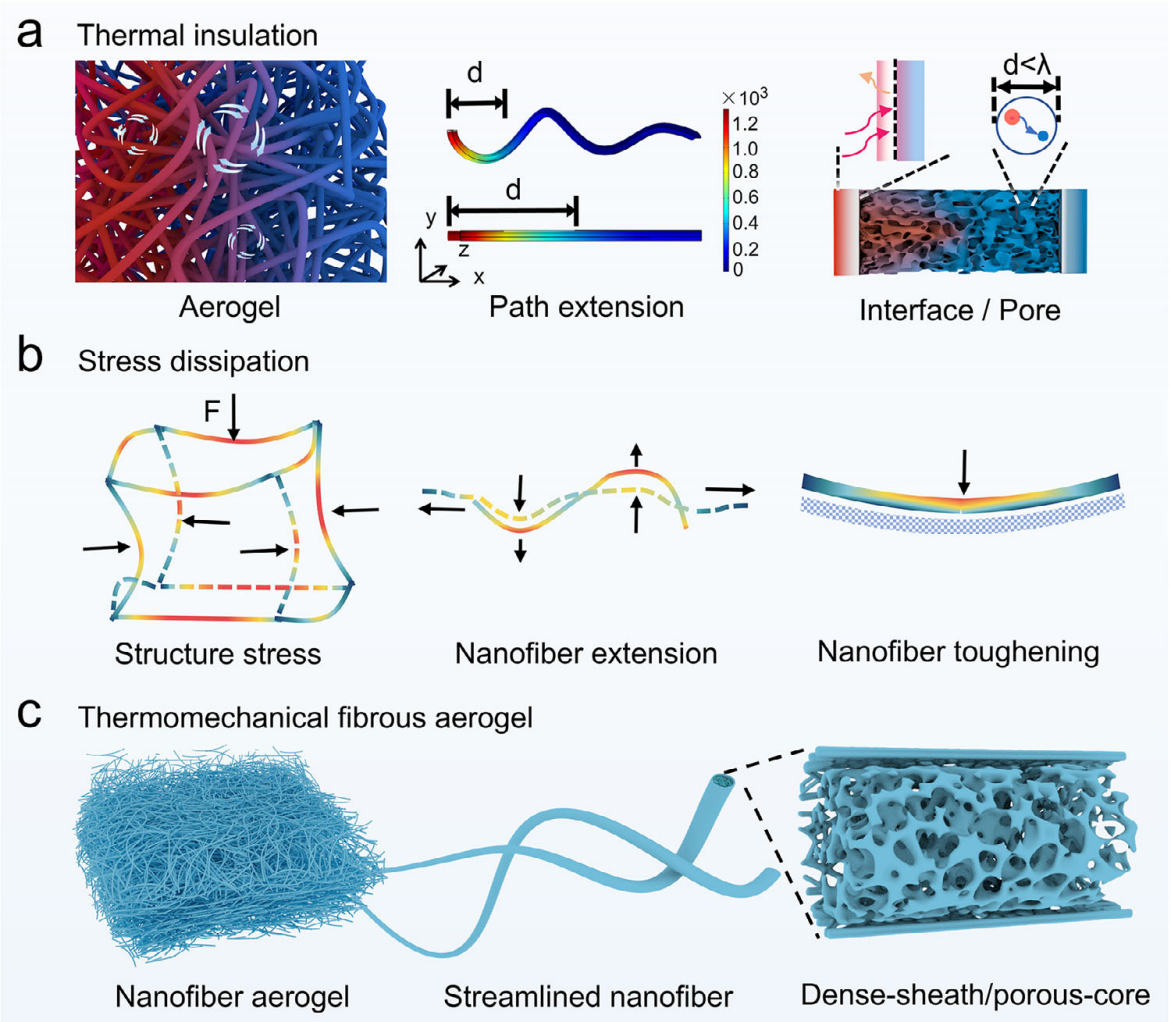
Structural design of ceramic fibrous aerogel toward thermal protection with thermomechanical stability, illustrating a) thermal insulation, b) stress dissipation. c) Structural schematic of the streamlined dual‐oxide fibrous aerogel.

Structural design of ceramic fibrous aerogel toward thermal protection with thermomechanical stability, illustrating **Scheme** **1**a,b. The structural schematic of the streamlined dual‐oxide fibrous aerogel is shown in Scheme 1c.

### 2.2 Structure and Morphology

**Figure 1:**
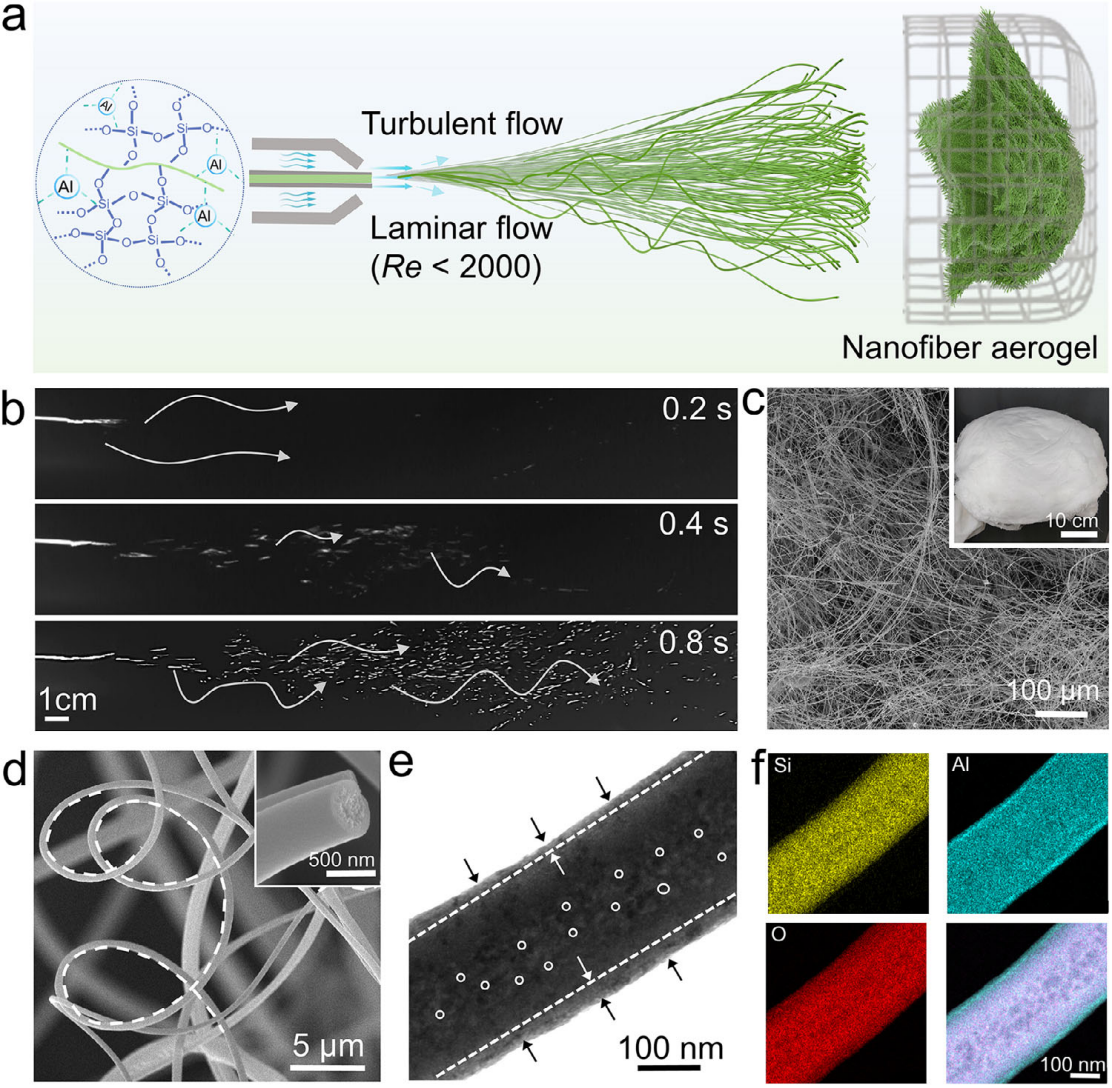
a) Schematic of streamlined dual‐oxide fibrous aerogel. b) Photos of the motion trajectory upon airflow in (a). SEM images of nanofiber aerogel at c) low‐magnification and d) high‐magnification. The inset in (c) is the photo of aerogel, and the inset in (d) is the cross‐section of one representative nanofiber. e) TEM image and f) elemental mappings of one nanofiber. The arrows, dashed line, and circles in (e) highlight dense‐sheath/porous‐core.

This work has independently set up the air flow spinning equipment for the expeditious fabrication of micro/nanofibers.^[^ ^33^ ^]^ As shown in **Figure** **1**a. The obtained solution was drawn into a syringe and mounted on a propeller. The spinning solution was squeezed through the 21G‐nozzle needle at a speed of 20 mL·h^−1^.^[^ ^34^ ^]^ Certain weak acids, exemplified by acetic acid, can serve as a copious source of H^+^ for fibrous aerogel.^[^ ^35^ ^]^ Leveraging the proton‐donor effect, these acids facilitate the formation of a dense network of hydrogen bonds within the fibrous aerogel. This facilitates the arrangement and enrichment of the required elements on the nanofibers. Subsequently, the desired structure can be formed. The formation of the structure is partly attributed to the manufacturing process. Due to the relatively low Reynolds number (*R*e) within the confined pipeline (less than 2000), the liquid exhibited laminar flow.

(1) $$Re=\frac{du\rho }{\mu }$$

where *R*e is the Reynolds number, dimension is 1; *d* is the diameter of the pipe (m), *u* is the velocity of flow (m·s^−1^), *ρ* is the density (kg·m^−3^), *µ* is the stickiness (m·Pa·s).

At the air‐liquid interface, the airflow‐induced shear is used to refine the solution extruded from the needle tip.^[^ ^36^ ^]^ The pressure of the outgoing air is controlled within the range of 0.1–0.3 MPa. The viscous droplets are stretched into fibers by high‐velocity airflow. At this moment, the gas flow field is turbulent. Just like Figure 1b, the jet rapidly evaporates within the high velocity airflow field and is solidified into streamlined nanofibers with the assistance of a heater. The fluctuations in airflow and disturbances induced by hot gases give rise to a variety of bending configurations in nanofibers within the flow field. The multi‐flow interactions intensify the formation of structures. A barrel‐shaped receiver with an average mesh size is positioned 50 cm away from the nozzle. Inside the receiver, the nanofibers continuously interweave and accumulate, ultimately forming a 3D nanofiber aerogel with a randomly entangled structure, as shown in the photo of Figure 1c. Then, through a folding process, the aerogel can be independently folded into the desired shape and structure. The final product can be formed by combining with a sintering process. During the sintering process, gradient calcination is employed. The temperature is initially raised from room temperature to 200 °C at a heating rate of 2 °C·min^−1^. Subsequently, it is slowly increased at the rate of 1 °C·min^−1^ until reaching 600 °C. Finally, the temperature is elevated to 900 °C at 3 °C·min^−1^ and maintained at this level for 2 h. This process is particularly crucial for the control of shaping and density.^[^ ^37^ ^]^

The scanning electron microscope (SEM) shows the structural characteristics of the nanofiber at different hierarchical levels, and the results confirm the original design. As shown in Figure 1c, fibrous aerogel consists of nanofibers with varying degrees of curvature that are randomly entangled and interconnected to form a 3D structure. The nanofibers are all in an amorphous form (Figure S1, Supporting Information). It also contains micro‐ and nano‐voids of varying sizes. This type of cross‐linking enables the aerogel to exhibit a dense 3D shape on the macroscopic scale. Internally, the pores are interconnected and self‐assembled, creating an unbounded space. Focusing on the fibers themselves, in Figure 1d, they are formed under the flow of an air field and have a high degree of curvature. These streamlined nanofibers produced in the turbulent flow field show distinct differences from the orderly oriented nanofibers produced in an electrostatic field.^[^ ^38^ ^]^ The disorder within the flow field induces a wider range of nanofiber deformations.^[^ ^39^ ^]^ The streamlined nanofibers provide strong support for the entangled structure, generating a more extended conduction path. Further magnification of the cross‐section of a single nanofiber reveals an apparent hierarchical structure with distinct inner and outer layers. The outer layer is relatively dense, while the inner layer is more porous.

For further analysis of the hierarchical structure, the images of the transmission electron microscopy (TEM) are shown in Figure 1e. There is a clear boundary between the inner and outer layers of the nanofiber. Here, the pores in the center of the porous nanofibers are more noticeable, and this structure also corresponds to the bionic principle mentioned above. Elemental analysis of Figure 1f shows that it has a structural feature in which Si is encapsulated by Al on the outside sheath. Comparing the properties of SiO_2_ and Al_2_O_3_, this structural distribution gives the nanofibers excellent mechanical and thermal insulation properties.

### 2.3 Mechanical Properties

**Figure 2:**
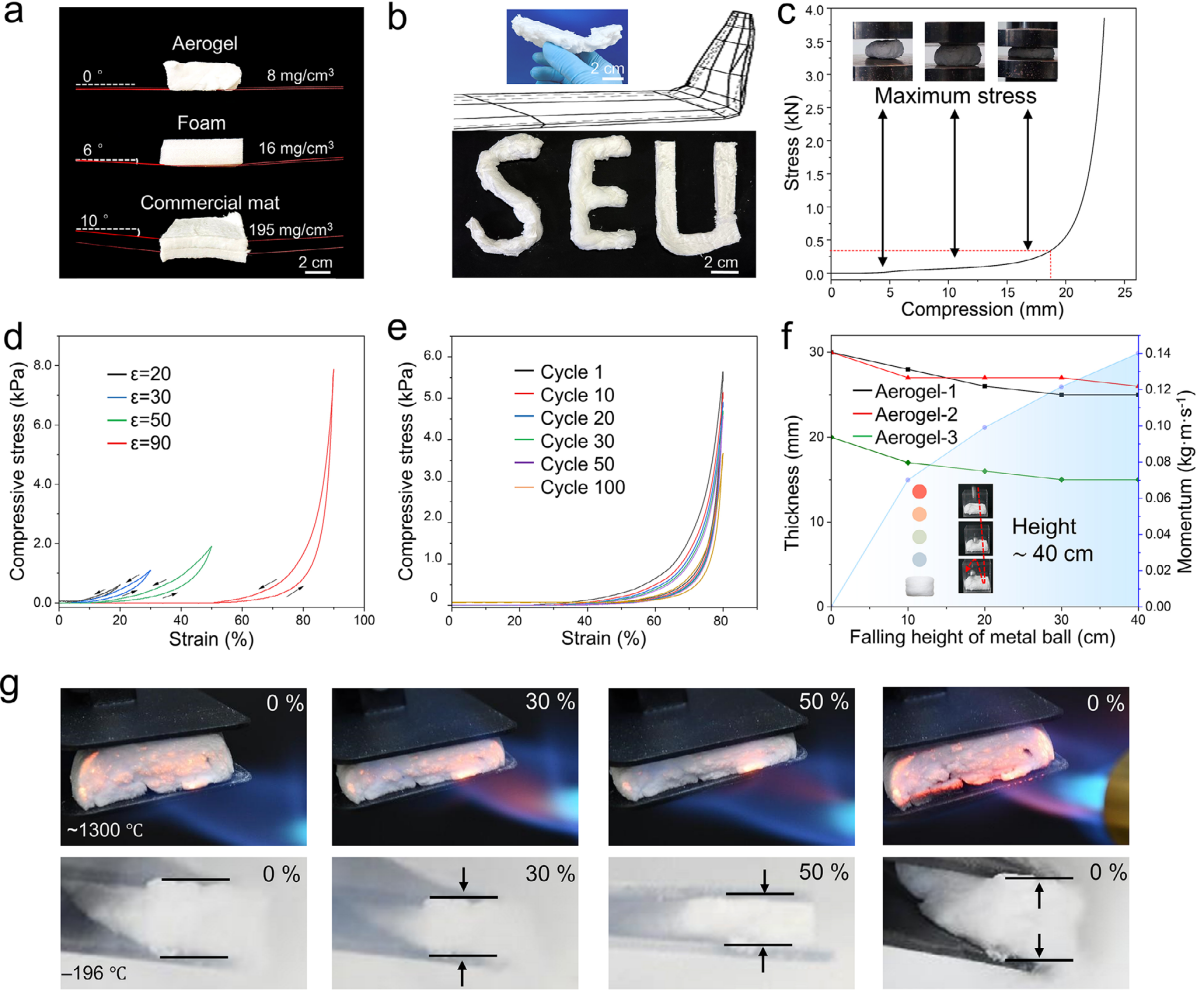
a) Comparison of weight and density among streamlined fibrous aerogel, commercial foam, and commercial fiber felt. b) Bending test and shape adaptability of aerogel bundles and aerogel blocks. c) Maximum stress test. d) Cyclic testing with different compression ratios. e) The compressive stress–strain curves at 80% strain for 100 cycles. f) Snapshot of drop impact test, and middle depression depth of different streamlined fibrous aerogel, under the change of falling height (change with momentum). g) Compression test at a hash temperature of 1300 and −196 °C.

With excellent dimensional stability and conformability, its light weight is particularly noteworthy. In **Figure** **2**a, the minimum density of fibrous aerogel can reach 8 mg·cm^−^^3^. The samples were selected to have similar volumes, with their dimensions ≈80 mm × 80 mm × 25 mm. All dimensional errors were within 3 mm. By placing fibrous aerogel, foam, and commercial mat simultaneously on a horizontal thin wire for comparison, it can be observed that fibrous aerogel does not cause any change in the thin wire, being as light as a feather. Its low density makes it extremely useful in the aerospace, automotive, protective clothing, and related industries.

Select a streamlined dual‐oxide fibrous aerogel of different proportions and sizes for bending tests in Figure S3 (Supporting Information). Shaping can be carried out before and after calcination. The shaping of nanofiber aerogel with excellent plasticity can be realized from multiple aspects. Shape fixation can be conducted prior to segmental burning, enabling the direct formation of the desired configuration. In addition, a two‐step calcination process—initial calcination followed by shaping and a secondary calcination—can be employed to enhance the stability of the resulting structure. Meanwhile, the flexibility and conformability of nanofiber aerogel can be leveraged for mold‐based shaping. It is observed in Figure 2b that a streamlined fibrous aerogel can form the desired shape according to the setting, demonstrating excellent flexibility. After multiple arbitrary bends, the slender nanofiber bundle can still be restored to its original shape. The amorphous structure formed by SiO_2_‐Al_2_O_3_ imparts toughness, and the high‐aspect‐ratio nanofibers achieved through spinning confer high mechanical strength.^[^ ^40^, ^41^ ^]^ The hierarchical pores inside and outside also enhance the plasticity of the nanofibers.^[^ ^42^ ^]^ The high curvature of nanofibers formed within the flow field exhibits superior cushioning performance under stress.^[^ ^43^ ^]^ Subsequently, the mechanical robustness of the aerogel arises from a synergistic effect.^[^ ^44^ ^]^ The fibrous network retains the benefits of its constituent nanofibers, while the high porosity provides a mechanism for excellent deformation via nanofiber slippage under stress.^[^ ^45^ ^]^ The same test method was then used to perform a bending test on the large‐scale fiber block. All results demonstrated the excellent flexibility and adaptability of the streamlined dual‐oxide fibrous aerogel. This is very useful for the formation of structural components.

This work then focused on testing the mechanical properties of streamlined dual‐oxide fibrous aerogel, such as compressive and impact strength. First, the performance was examined in the compression test. Dimensional stability is a relatively important indicator for nanofiber aerogels. Once subjected to a pressure of up to 300 N, fibrous aerogel can suffer destructive damage. In Figure 2c, the maximum pressure it can withstand is 125 kPa. The compression cycle test was carried out again. The repeated tests of Figure 2d were performed by continuously adjusting the compression ratios to 20%, 30%, 50% and 90%. It was found that the nanofiber aerogel could still recover after being compressed downward hundreds of times. The streamlined dual‐oxide fibrous aerogel underwent long‐term cyclic tests under extreme compression, compressing it by 80% each time. It was found that extreme conditions would cause some damage to the nanofiber aerogel. The streamlined dual‐oxide fibrous aerogel retained a good structure over dozens of cycles in Figure 2e, demonstrating excellent fatigue resistance. But the thickness inevitably decreased after prolonged compression. In the absence of any scale‐up design of the structure, this excellent compression resistance is due to the structural characteristics of the nanofibers themselves. The streamlined nanofibers with a hierarchical structure give the network good deformability.

To further investigate the mechanical properties of fibrous aerogel, it was subjected to an air drop impact test, just like Figure 2f. A 50 g solid metal ball is allowed to fall freely from various heights and eventually hits the nanofiber aerogel below. After absorbing the impact, fibrous aerogel only has a small dent on its surface, maintaining the integrity of its overall morphology. Observing the moment of contact between the ball and the aerogel, it can be seen that the ball rebounds after hitting the fibrous aerogel until it loses all its energy and finally comes to a standstill. The specific details can be seen in Figure S4 (Supporting Information). When the ball falls, the gravitational potential energy is converted into kinetic energy. The velocity and kinetic energy it acquired will generate an impact force on the nanofiber aerogel, which can be described in terms of momentum. Varying the dropping height of the ball: the higher the height, the greater the momentum. Repeating the above‐mentioned experimental operations, fibrous aerogel with different densities and degrees of compactness was simultaneously compared. By measuring the height of the central dent, it can be seen that the deformation of the nanofiber aerogel is limited, demonstrating its excellent impact‐resistance performance.

The material's resistance to external forces under extreme temperature conditions must be tested, and its stability in complex environments must be studied. The experiments were conducted by subjecting the samples to thermal shock at an elevated temperature of 1300 °C and a cryogenic shock using liquid nitrogen at −196 °C. A compression experiment was conducted in conjunction with the exposure of the subject to extreme temperatures. The image is presented in Figure 2g. It has been demonstrated that, in such circumstances, the streamlined dual‐oxide fibrous aerogel retains its resilience characteristics and maintains its intrinsic structure. This finding suggests that the material demonstrates exceptional stability under extreme temperature fluctuations, which renders it a promising candidate for utilization in force‐heat coupling environments.

### 2.4 Thermal Management and Mechanical‐Thermal Coupling

**Figure 3:**
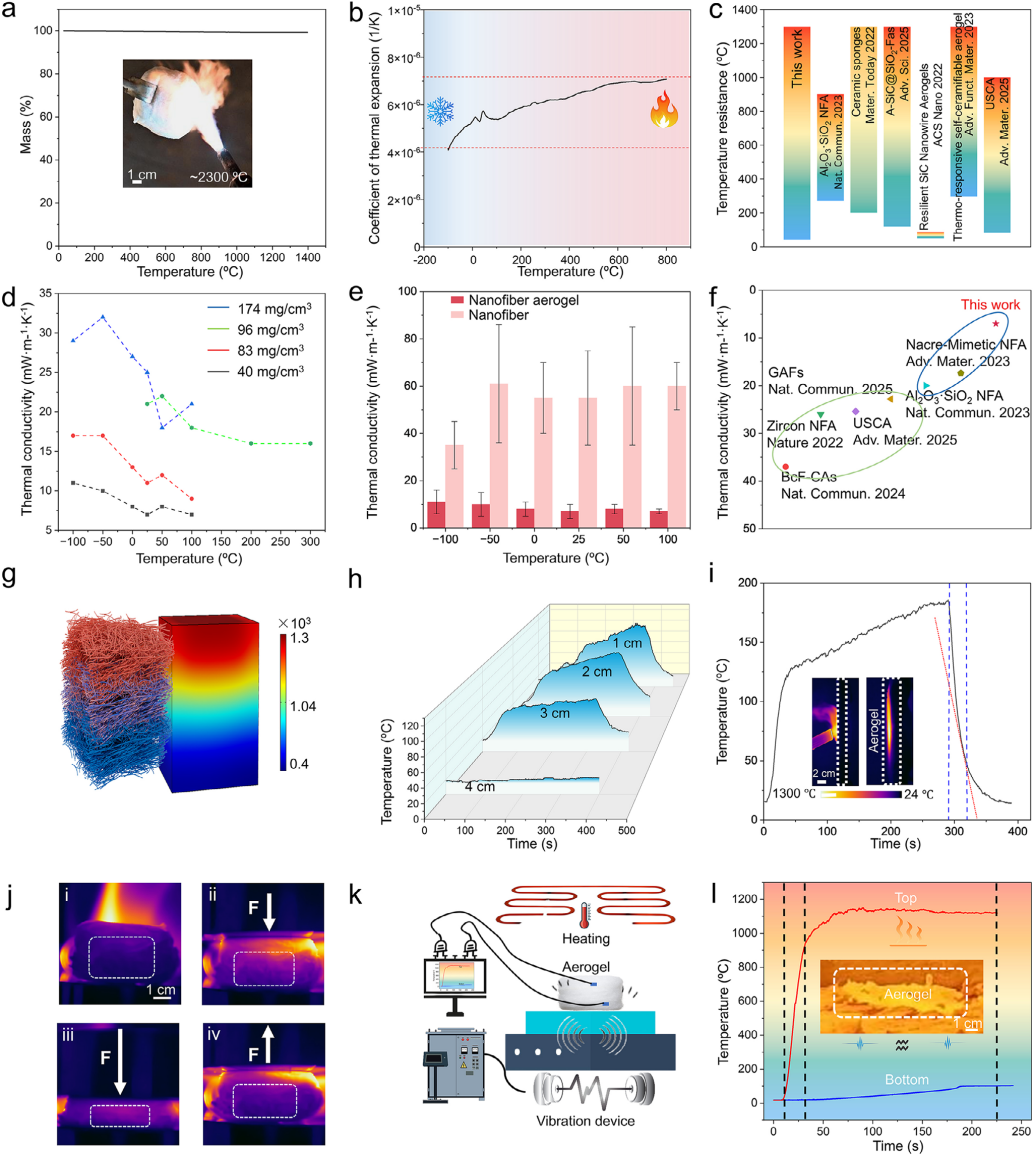
a) TGA curve of fibrous aerogel. The inset is the picture of a flame retardant upon an acetylene torch. b) Curve of coefficient of thermal expansion from −100 to 800 °C. c) Comparison of insulation temperature range between fibrous aerogel and recent reports. d) Thermal conductivity of aerogel with different densities. e) Comparison of thermal conductivity between aerogel and powder counterpart. f) Thermal conductivity between fibrous aerogel and recent reports. g) COMSOL simulation showing the heat diffusion within nanofiber aerogel. h) The impact of aerogel thickness on thermal insulation. i) Curve of heat dissipation. The inset is the thermal image of thermal insulation upon a butane spray gun. j) Thermal insulation capability upon compression at 1300 °C. k) Schematic of simulated space‐ground integrated test. l) The temperature curve at the top and bottom of the aerogel tested in (k). The inset is the photo of aerogel during the test in (k).

Thermal insulation is the most common application scenario for inorganic nanofiber aerogels. **Figure** **3**a illustrates the thermal stability of the material at high temperatures. The curve was flat within the temperature range of 20–1400 °C, whereas the mass of fibrous aerogel exhibited almost no obvious change. The test of the acetylene torch shows its flame‐retardant properties at extremely high temperatures (≈2300 °C). And the material has an extremely low (even negligible) coefficient of thermal expansion, as in Figure 3b. This indicates its ability to maintain optimal structural stability at elevated temperatures. All curves and tests indicated that the prepared materials had superior thermal stability. The thermal insulation property of fibrous aerogel was tested using a butane blowtorch. An infrared thermal imager was used to monitor the temperature attained on the material's posterior surface when subjected to a temperature of 1300 °C. When subjected to rapid thermal shock from extremely high temperatures, the temperature of the material's rear surface increases gradually. Upon attaining a maximum, the temperature then remains relatively constant. This performance is comparable to the best values reported in the extant literature. The comparison of the insulation temperature range is shown in Figure 3c. Thermal conductivity is a key indicator of performance. Nanofiber aerogel has a unique porous structure with high porosity and a low proportion of solid skeleton. Most of the internal volume is filled with gas, and the thermal conductivity of air is much lower than that of solid materials. This results in a significant reduction in overall thermal conductivity.^[^ ^41^ ^]^ The streamlined nanofibers designed in this work further enhance this advantage. The double hierarchical pores suppress the phonon transport of the nanofibers and the interfacial thermal resistance. Their infinitely extended paths effectively reduce solid‐state heat conduction. The randomly entangled structural network also restricts the movement of gas molecules, inhibiting heat convection transfer. Figure 3d measured the thermal conductivity of streamlined dual‐oxide fibrous aerogel at different densities and temperature variations. During the testing process, the compression that occurred resulted in a measured density that was greater than the actual density. According to the definition of thermal conductivity, the lower the density, the lower the thermal conductivity.^[^ ^46^ ^]^ Even so, the lowest thermal conductivity of streamlined dual‐oxide fibrous aerogel at room temperature can reach 7 mW·m^−1^·K^−1^. (This greatly exceeds the thermal conductivity of air, and has a significant advantage over most currently reported ceramic aerogels.) Figure 3e compares the thermal conductivity of aerogel and dense powder. And the results demonstrated consistent performance across different sample batches. This reflects the advantage of the aerogel structure. Compared with the relevant reports in Figure 3f, it can be found that the thermal conductivity in this work is excellent.^[^ ^47^, ^48^ ^]^ Increases in the thickness of the fibrous aerogel result in a decrease in the temperature on the opposite side of the heat source. Figure 3g indicates that after the thickness exceeds the size limit, thereby showing an extremely strong thermal insulation effect.^[^ ^49^ ^]^ As shown in Figure 3h, it is notable that at a thickness of 4 cm, the temperature on the opposite side can be maintained at room temperature. The material exhibits a comparatively diminished specific heat capacity. Upon cessation of the heat source, the material undergoes an effective cooling process in Figure 3i. This is its property of dissipating heat with considerable rapidity. The diagram of the comparison effect is shown in Figure S5 (Supporting Information). The thermal imaging pictures are intended to provide a visual demonstration of the aforementioned data. Following a 5 min heating process, a substantial temperature difference between the surfaces of fibrous aerogel was attributed to its exceptional thermal insulation and protective capabilities. Further testing of its thermal insulation performance was conducted under the coupling of force and heat, showing that it still exhibited good thermal insulation stability in Figure 3j.

Furthermore, we carried out more realistic tests to investigate its practical applications. The thermal‐vibration test apparatus was independently constructed by our institution, as shown in Figure S6 (Supporting Information). It was utilized to simulate the thermal shock and vibration environment in the flight scenario, which is the integrated space‐ground test. The conceptual diagram of the device is shown in Figure 3k. The data was collected using thermocouples positioned above and below the material.^[^ ^50^ ^]^ In the rapid heating thermal shock and on the long‐term vibration platform, the material showed excellent thermal and structural stability. The temperature rise on the underside of the material was extremely low, and the overall structure did not deform, just like Figure 3l. This indicates that the material maintains stable performance in complex environments and has already demonstrated practical value in real‐world applications such as mobile, flight, and other motion scenarios.

### 2.5 Multifunctional Applications

**Figure 4:**
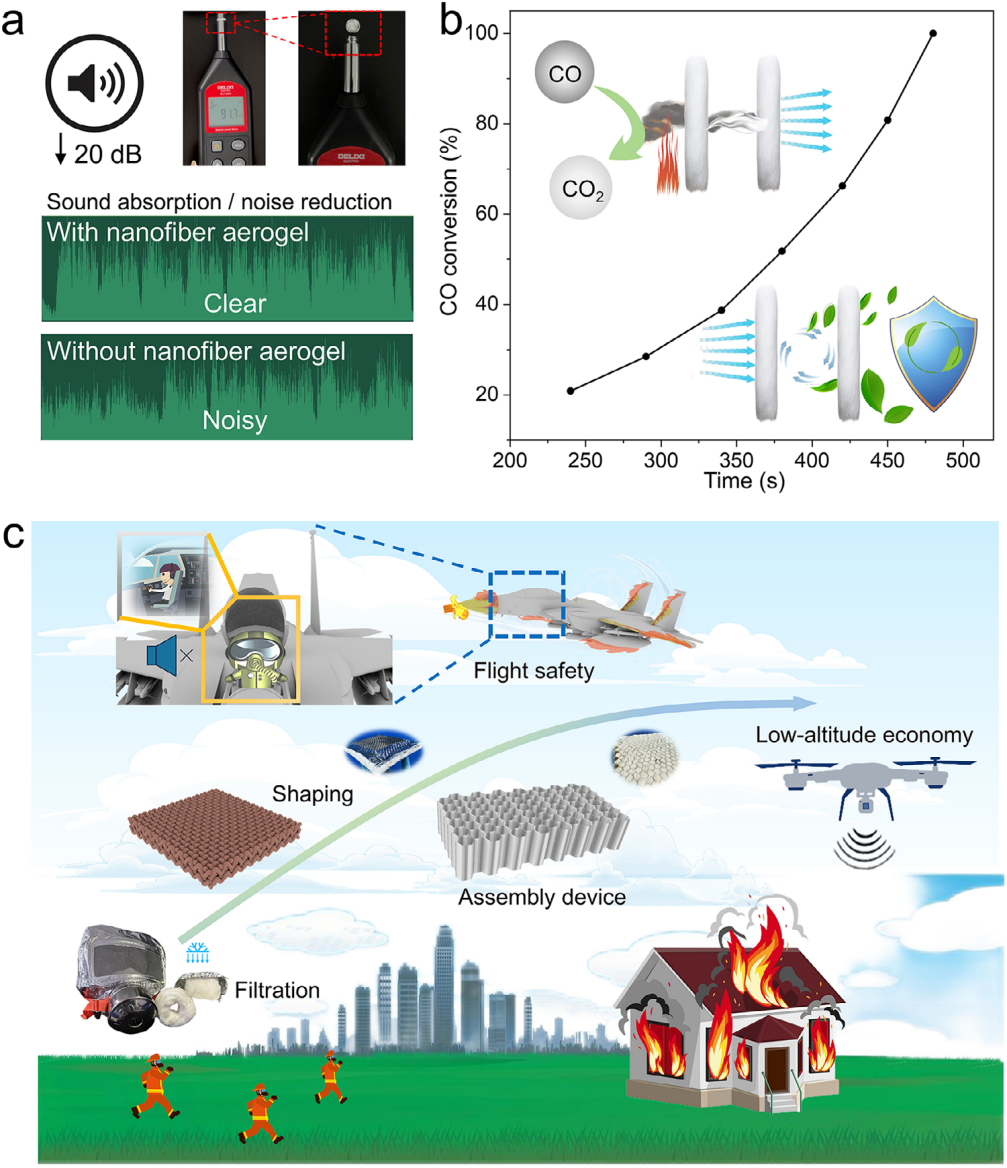
a) Performance tests of sound absorption and noise reduction. b) The conversion of CO shows the capability of protecting personal safety under fire smoke. c) Schematic illustration of aerogel shaping and device assembly for the application scenarios from sky to ground.

Depending on its own structural and functional characteristics, fibrous aerogel can achieve multifunctional development and utilization. As shown in **Figure** **4**a, data acquisition was performed by a decibel test before and after covering the sound source with nanofiber aerogel. Different test data of the same type were shown in Figure S7 (Supporting Information). This can reduce the volume by ≈20 dB. The sound insulation performance was significantly enhanced with increasing thickness. Furthermore, nanofiber aerogels with a higher density exhibited superior acoustic attenuation. The comparison is shown in Figure S10 (Supporting Information). Meanwhile, the 3D nanofiber aerogel can make the sound clearer. The 3D oxide nanofiber aerogels have a porous structure that can isolate some gases and thick smoke.^[^ ^51^ ^]^ Meanwhile, compared with other composite materials, it has the advantage of oxygen vacancies and can be combined with a small amount of elemental loading.^[^ ^52^ ^]^ That can convert carbon monoxide into carbon dioxide, further reducing the damage of the fire scene to the human body, as shown in Figure 4b. In case of explosion and other hazards, the strong mechanical properties and flexibility of nanofiber aerogel can provide protection. The specific functional display is shown in Figure S8 (Supporting Information).

In the future, it is hoped that the unique properties of fibrous aerogel will enable more applications in different scenarios, achieving success in both ground and space exploration applications. Material shaping and device assembly will be carried out, they are shown in Figure 4c. It will ensure the safety of the aircraft and internal personnel during high‐speed operation. The characteristics of light weight and high temperature resistance make it applicable in high temperature and load‐bearing environments. Its excellent performance enables it to provide significant assistance in the current low‐altitude economy, realizing the connection between heaven and earth. Regarding fire safety, it can protect people in fire scenes from the invasion of flames. In addition, its capriciousness allows it to be used in protective clothing and structures in various aspects. This helps to protect lives and property in both work and life.

## 3 Conclusion

In summary, the 3D nanofiber aerogel composed of double‐layered porous streamline nanofibers possesses excellent flame retardancy and thermal insulation. The multi‐layered thermal insulation structure formed by random winding of this unique structure endows the nanofiber aerogel with extremely low thermal conductivity, while maintaining structural integrity and ensuring good mechanical properties. The flexibility and plasticity of fibrous aerogel enable it to adapt to different application scenarios and be integrated into various complex engineering systems. Structural optimization and design have resulted in diverse hierarchical structures of streamlined dual‐oxide fibrous aerogel. Thus, the development of 3D ceramic nanofiber aerogels, which consist of flexible fibers with structural properties and functional stability, will offer crucial guidance for future thermal management advancements. They promise more effective thermal insulation solutions for the aerospace, automotive, and industrial sectors. By optimizing structure and properties, researchers can anticipate technological breakthroughs and the emergence of more reliable and durable thermal‐protection systems. Owing to its lightweight, porous nature, and high‐temperature and corrosion resistance, this material can perform heat management, safety protection, sound insulation, and noise reduction. It is anticipated that this material will find applications in various aspects of social life, including flight scenarios, automotive safety, and firefighting clothing.

## 4 Experimental Section

### Materials

Polyvinyl pyrrolidone (PVP) (*M*_w_≈1300 000) was obtained from Sigma–Aldrich Co., Ltd. Polyvinyl Butyral (PVB) (*M*_w_ 90 000–1200 000) was obtained from Macklin Co., Ltd. Aluminum acetylacetonate, Hexadecyl trimethyl ammonium bromide (CTAB) were obtained from Aladdin Co., Ltd. Ethanol, Oxalic acid, Acetic acid, Tetraethyl orthosilicate were purchased from Sinopharm Chemical Reagent Co., Ltd. All chemical reagents employed in this work were of reagent grade and used without further purification.

### Blow‐Spinning Solution of Nanofiber Aerogels

Blow‐spinning liquid precursors were prepared by the sol‐gel method in a stepwise dissolution process.^[^ ^53^ ^]^ First, 3 g PVP and 1 g PVB were added to 30 mL of Ethanol and stirred for 12 h at room temperature,^[^ ^54^ ^]^ named A. Simultaneously, 0.04 g of Oxalic acid and 6.25 g of tetraethyl orthosilicate were dissolved in 20 mL of Ethanol and 2 mL of deionized water. The mixture was stirred continuously at 38 °C for 12 h. A homogeneous silica aqueous sol was obtained by hydrolysis and polymerization, named B. Later, 40 mL of Acetic acid, 9.73 g Aluminum acetylacetonate, and 0.3 g CTAB were added to B, and the mixture was stirred until it became clarified. Then, B was poured into A, and the resulting mixture was stirred for 4 h to ensure thorough homogenization. The relevant experimental steps are analogous to those previously delineated. Different densities and degrees of compactness could be achieved by adjusting the polymer template. The quantity of Acetic acid was contingent upon the ratio of aluminum to silicon. The comparison of different additive ratios is shown in Figure S2 (Supporting Information).

The spinning was carried out at a room temperature (25–30 °C) and low relative humidity (< 40%). The air pressure was controlled within the range of 0.1–0.3 MPa, with a pressure of 0.2 MPa predominantly used in this experiment. Notably, a positive correlation was observed between the applied pressure and fiber fineness, with higher pressures yielding finer fibers.

### Characterization

The morphology and microstructure of fiber aerogels were analyzed by scanning electron microscope (SEM, JSM‐6700F, Hitachi) and transmission electron microscopy (TEM, JEM‐2100F, Japan). The crystal structure was measured using the X‐ray diffractometer (XRD, Ultima IV, Japan). The thermal stabilities of the insulation materials were tested using a Simultaneous thermal analyzer (STA, STA449 F3, Germany), whereas the maximum temperature was 1400 °C in N_2_ atmosphere. The thermal conductivity was tested using a Flash thermal conductivity instrument (LFA, Netzsch lfa 467, Germany) and a Differential Scanning Calorimeter (DSC, Netzsch dsc 214, Germany). The temperature test was conducted using an Infrared radiation thermal imager (IRTI, FOTRIC 600C, China).

## Conflict of Interest

The authors declare no conflict of interest.

## Supporting information



Supporting Information

## Data Availability

The data that support the findings of this study are available in the supplementary material of this article.
