# Supplementary material for: All‐In‐One Thermal Protection: Multifunctional Synergy in Hierarchically Structured Dual‐Oxide Nanofiber Aerogel
Source: Adv Sci (Weinh). 2025 Nov 21;13(5):e16126. doi: 10.1002/advs.202516126 (PMC12850046; doi:10.1002/advs.202516126)
Supplement: Supplementary file 1 — Supporting Information [file ADVS-13-e16126-s001.docx]

Supporting Information

All-in-One Thermal Protection: Multifunctional Synergy in Hierarchically Structured Dual-Oxide Nanofiber Aerogel

*Zijian Zhao^‡^, Shujing Li^‡^, Han Ma, Zhou Zhou, Wentao Zhao, Zichen Wei, Yanbin Li, Qingguo Fei, Yueming Sun, Yunqian Dai^*^*

Z. Zhao, S. Li, Z. Zhou, W. Zhao, Y. Sun, Y. Dai

School of Chemistry and Chemical Engineering

Southeast University

Nanjing 211189, China.

E-mail: daiy@seu.edu.cn

H. Ma, Z. Wei, Y. Li, Q. Fei

School of Mechanical Engineering, Southeast University

Southeast University

Nanjing 211189, China.

Z. Zhao, S. Li, H. Ma, Z. Zhou, W. Zhao, Z. Wei, Y. Li, Q. Fei, Y. Dai

Ministry of Education Key Laboratory of Structure and Thermal Protection for High-Speed Aircraft

Nanjing 211189, China.


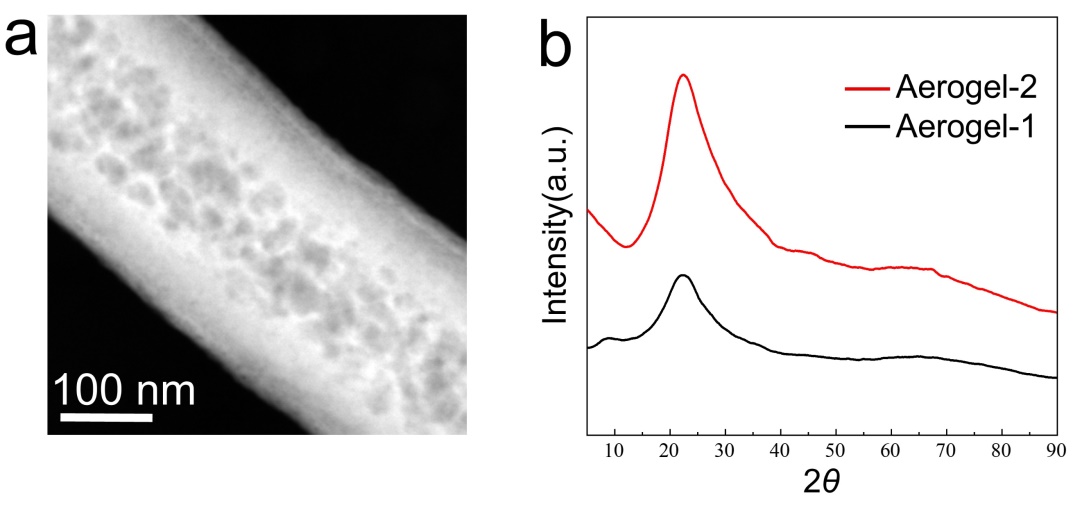


**Figure S1** (a) TEM image of a single dual-oxide fiber. (b) XRD patterns of different element ratios to prove the amorphous structure.


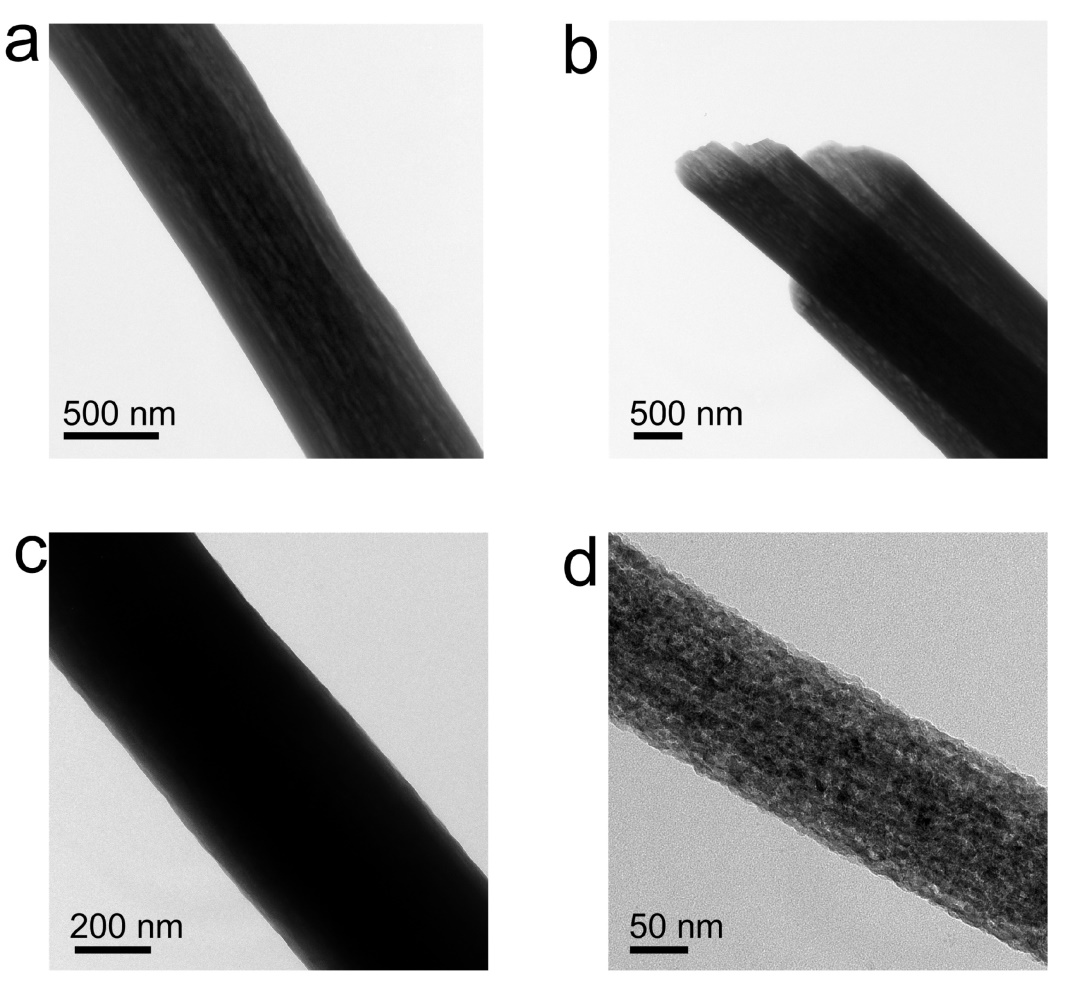


**Figure S2** TEM image of fibers with different components. (a) The order structure of SiO_2_-Al_2_O_3_, (b) core-shell structure of SiO_2_-Al_2_O_3_, (c) fiber of SiO_2_, (d) fiber of Al_2_O_3_. This is used to demonstrate the structural superiority and controllability of nanofiber aerogel in this work.


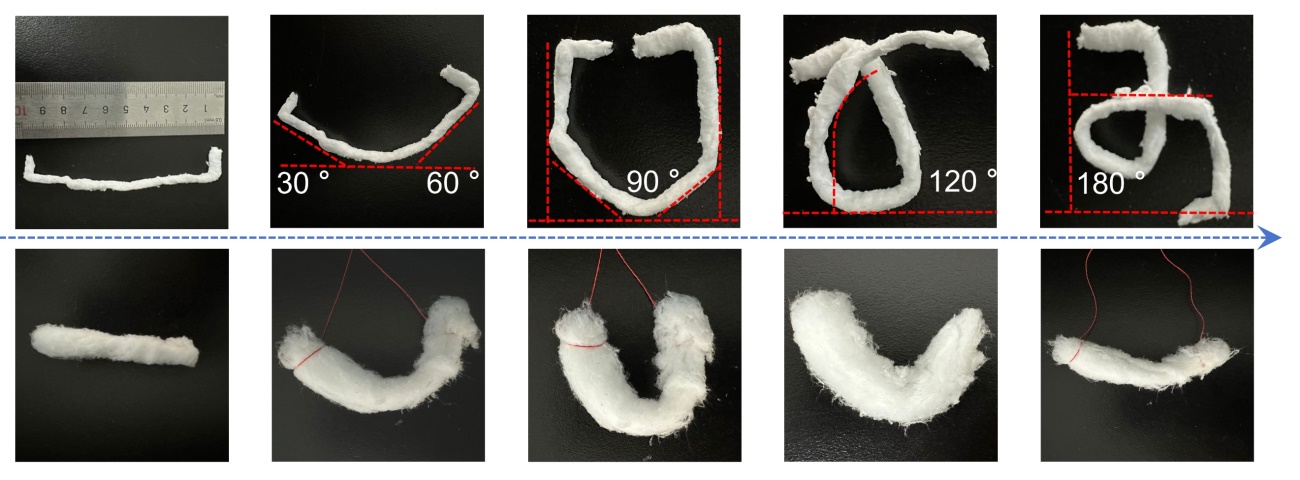


**Figure S3** The photo of the bending test for aerogel bundles and aerogel blocks**.** The bending degree ranges from 0 — 180 °. It indicates that the nanofiber aerogel is deformable and flexible.


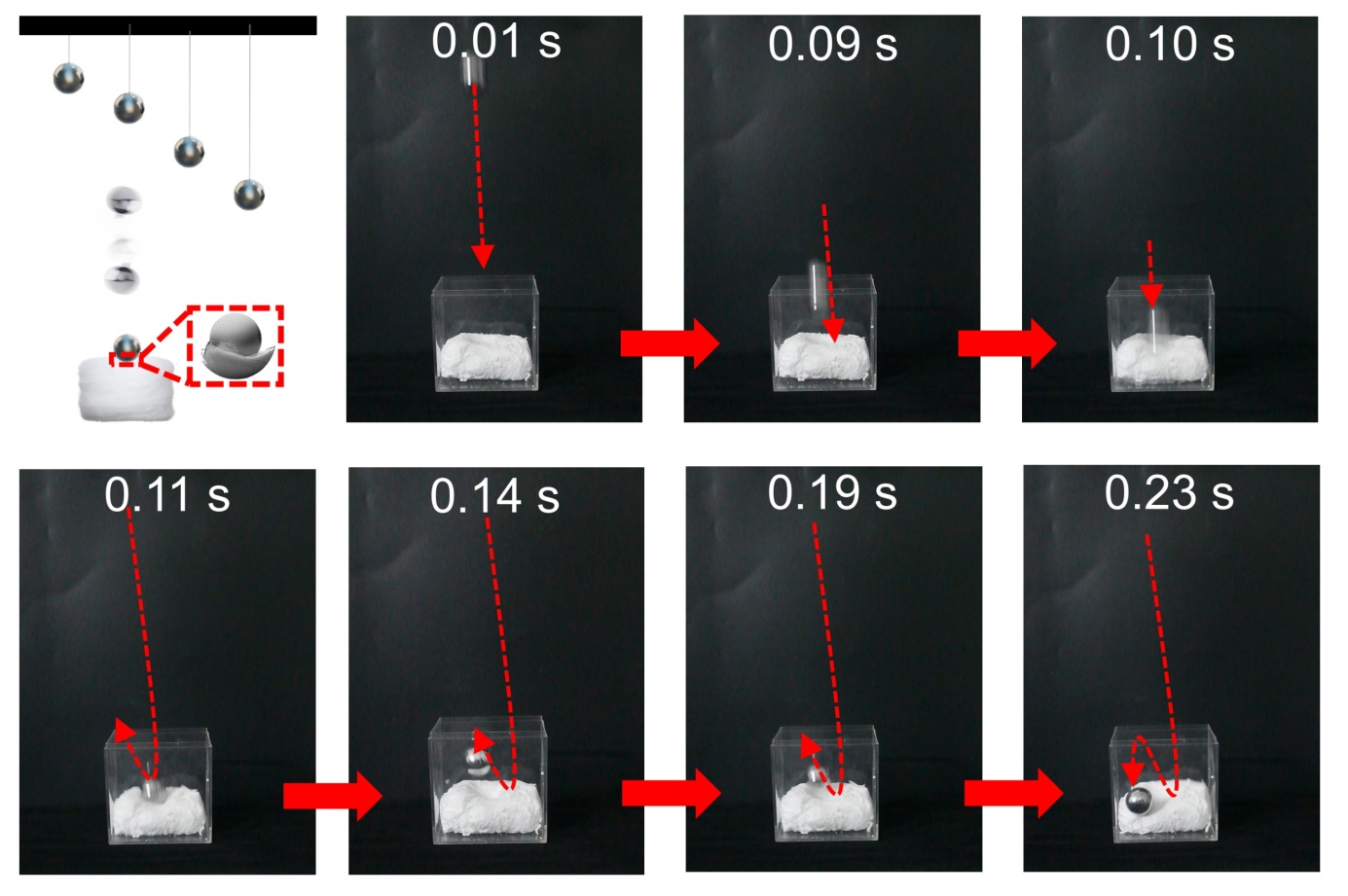


**Figure S4** Snapshot of impact experiment. Drop the ball from different heights to observe the impact situation. The picture shows the fall and rebound effect of the ball over time at a height of 20 cm.


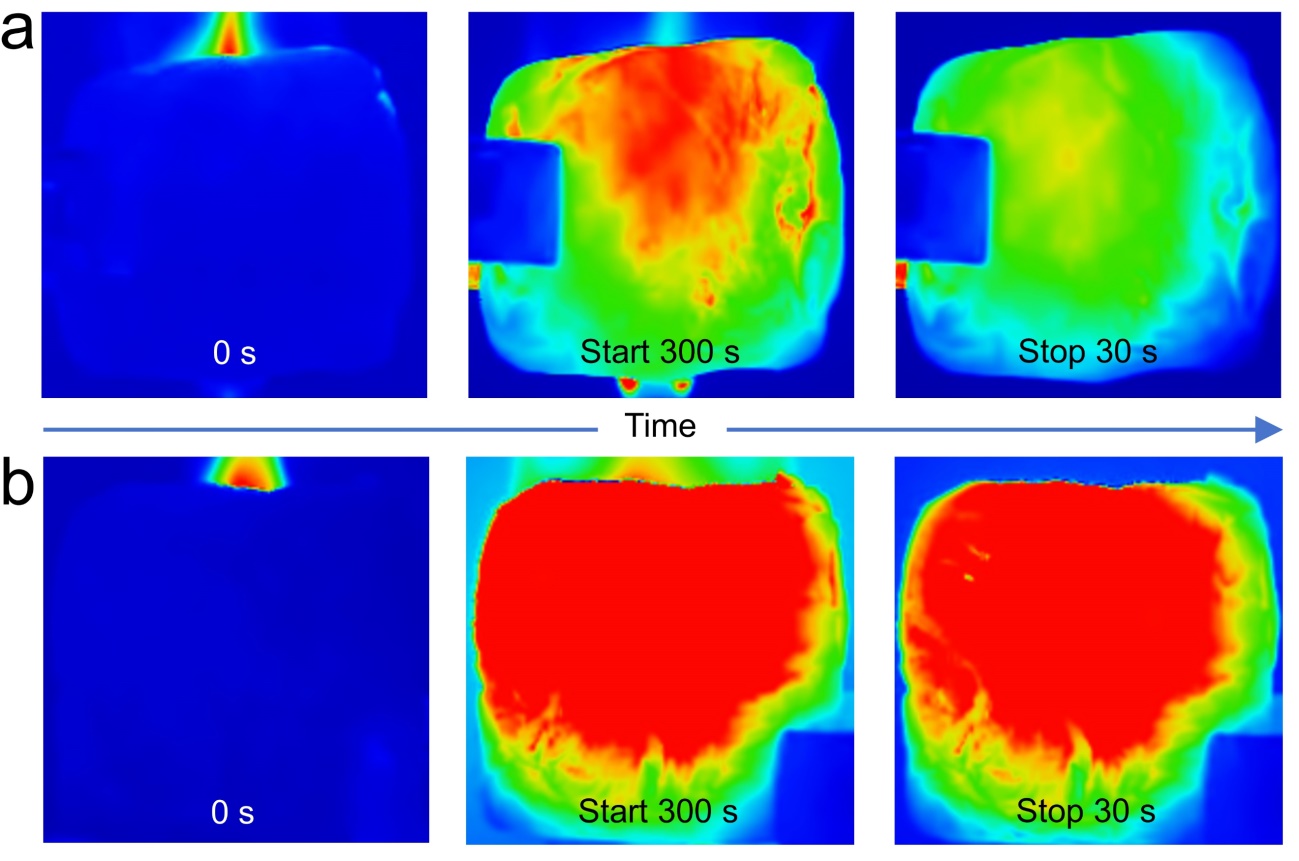


**Figure S5** (a) The image of quick heat dissipation for streamlined dual-oxide fibrous aerogel. (b) The image of heat dissipation for other fiber aerogel. This shows that the nanofiber aerogel of this work has super heat insulation ability and fast heat dissipation ability.


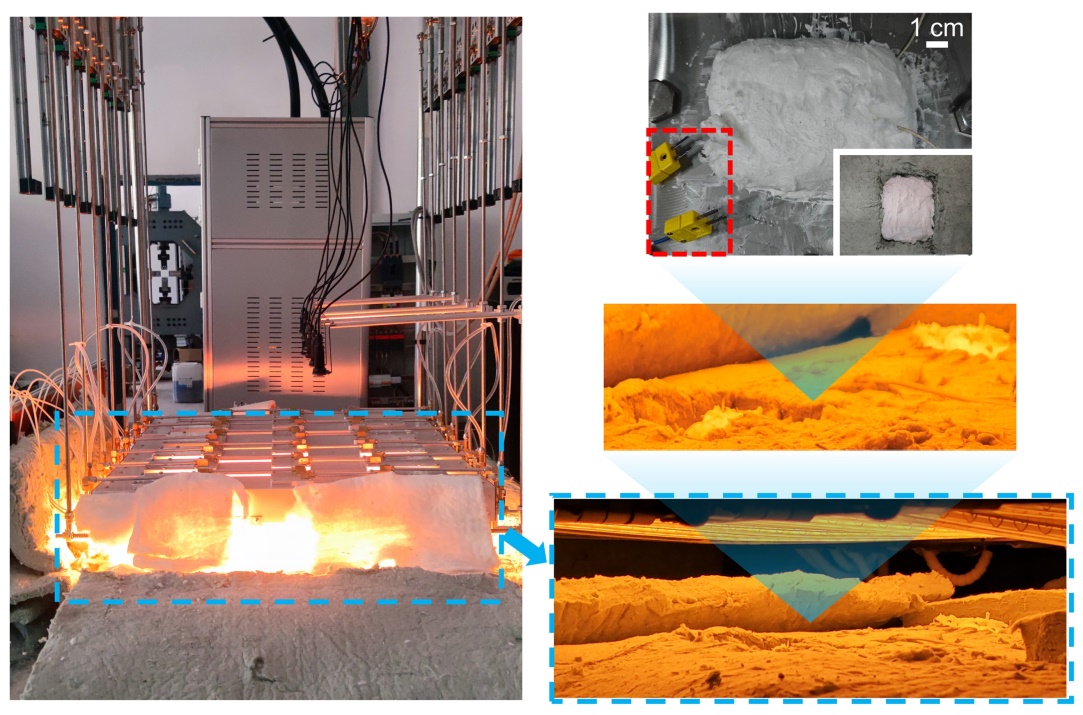


**Figure S6** The actual device of thermal vibration. The nanofiber aerogel module is placed between the vibrating platform and the quartz lamp to study the stability of performance under simulated space-ground integrated experiments.


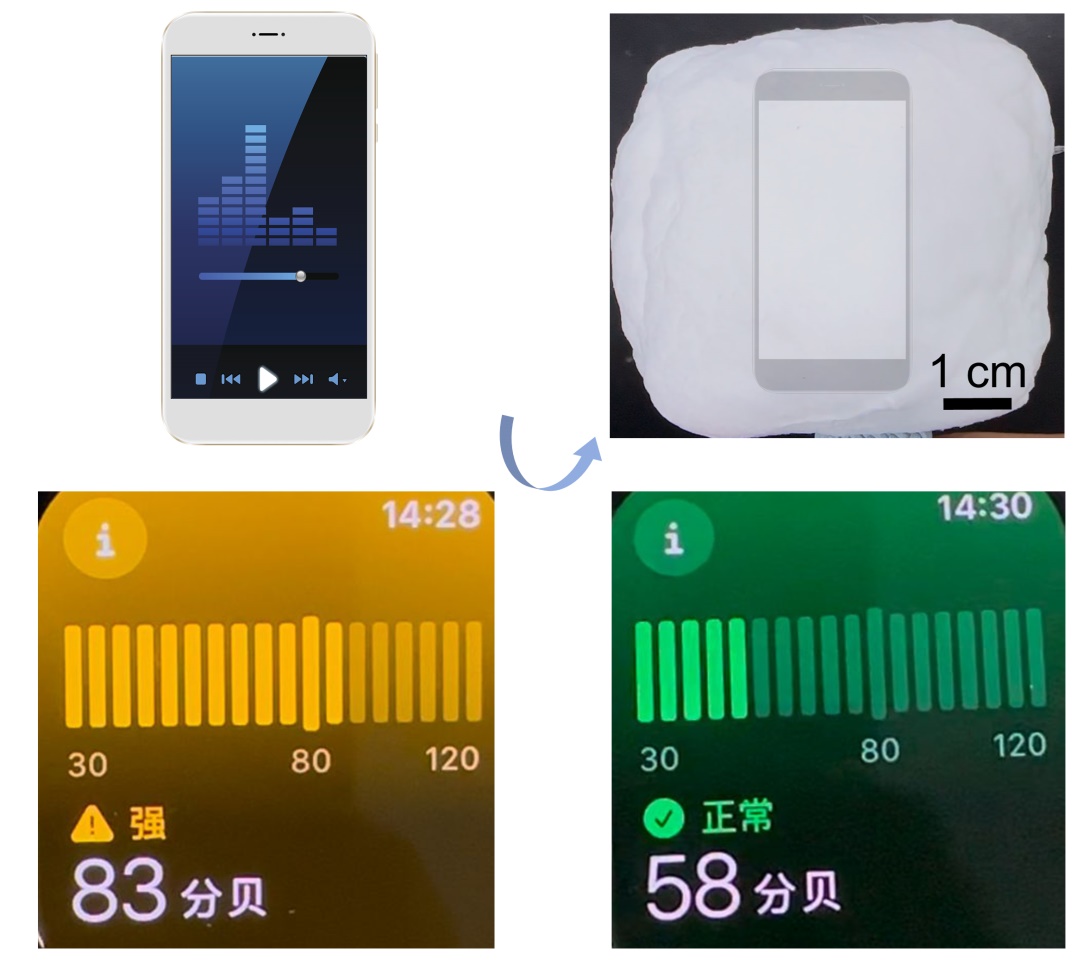


**Figure S7** The test of sound insulation showing that nanofiber aerogel has a certain sound absorption effect.


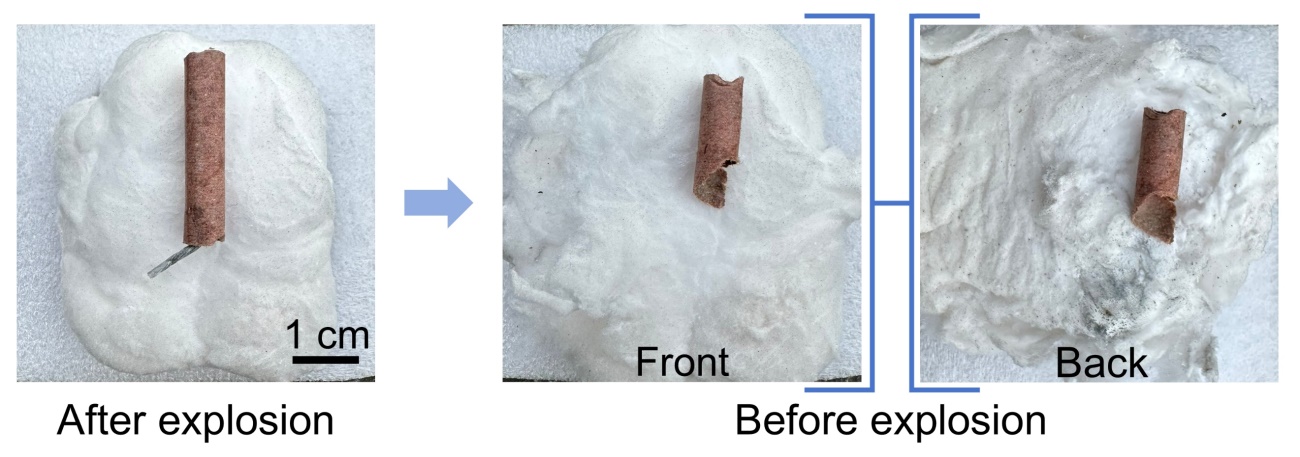


**Figure S8** The test of explosion showing that the super mechanical properties of nanofiber aerogel protect against the impact of an explosion.


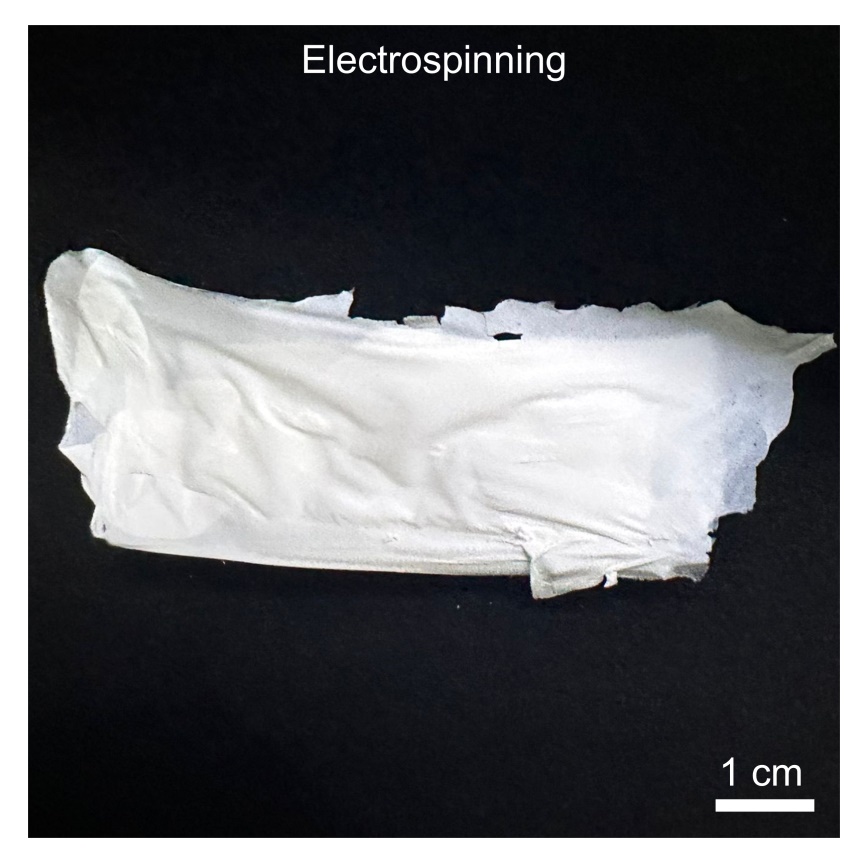


**Figure S9** The straight nanofiber prepared by electrospinning. The sharp contrast indicates the superiority of the process in this work.


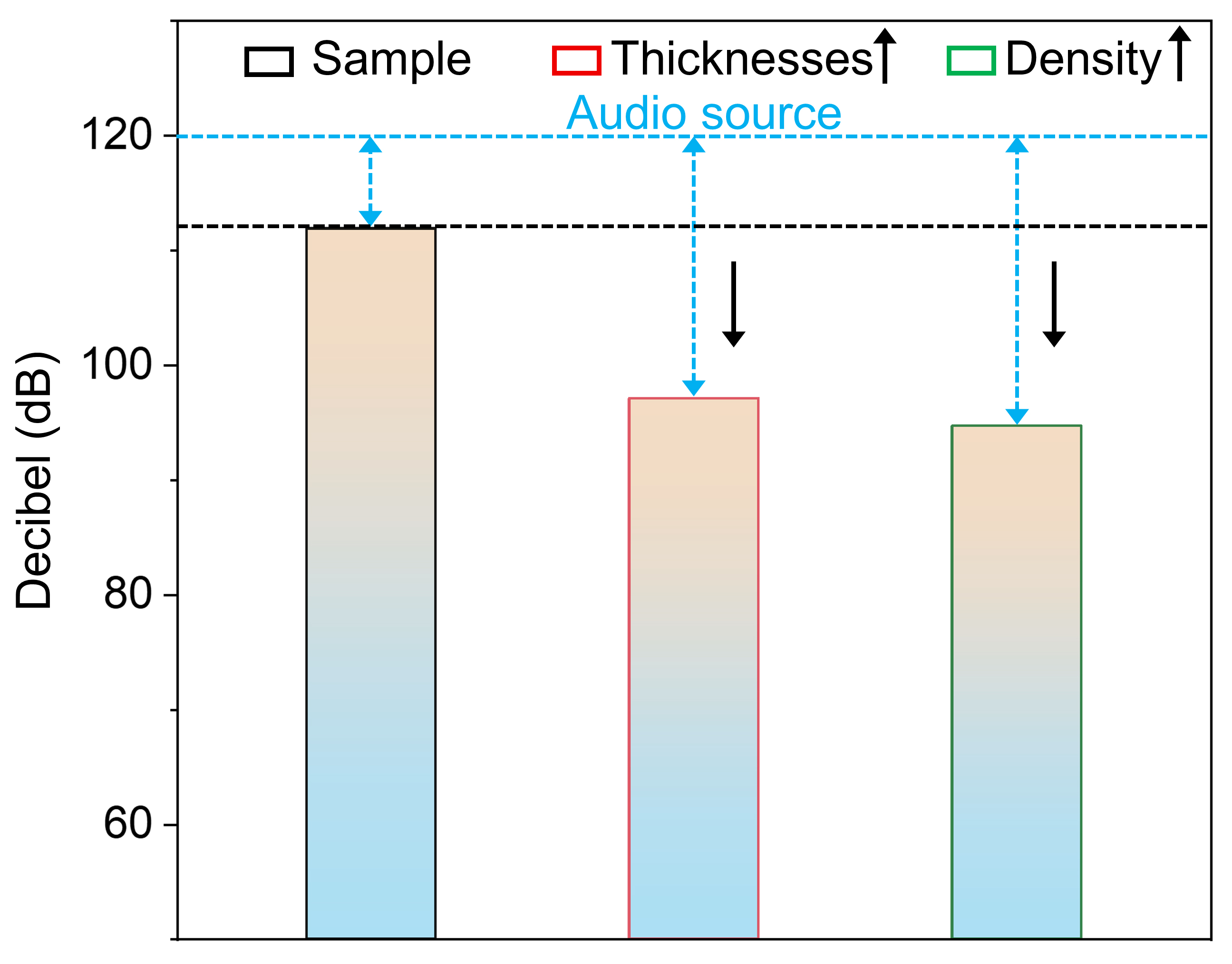


**Figure S10** A comparative study of the sound insulation properties was performed by varying the thickness (3 cm) and density (20 mg·cm^–3^) of the samples (1 cm, 8 mg·cm^–3^).
